# Chicago Medical Society

**Published:** 1882-02

**Authors:** 


### ARTICLE VIII.

CHICAGO MEDICAL SOCIETY. Dec. 5, 1881. Dr. E. Ingals, President, in the Chair.

### MALARIAL KERATITIS.

Dr. F. C. Hotz read a paper with the above title, and recorded a few cases from his notes and from memory. In ordinary cases of keratitis, he kept the eye shut, and covered up with charpie, retained in position by a bandage. Superficial ulceration could thus be made to heal very readily. In describing malarial keratitis, he said that the ulcer of the cornea is raised, and may creep on the entire surface; there is severe pain, photophobia and lachrymation. It extends for a few weeks, and reparation may require a few months. This takes place through an elongation of the deeper blood-vessels. This ulcer is just as characteristic as the patches found in the mouth in syphilis. Malarial or remittent fever may or may not accompany or precede this affection; as a rule it does, and quinine is especially successful as a constitutional treatment, even after local treatment has failed.

*Case* I.—Æt. twenty-six, came under treatment in January, 1881. Five days previous to this, patient had a chill. Keratitis set in, and a linear abrasion of the epithelium formed. He prescribed a lotion of atropine, and saw the case five days later. There had been no relief, and the inflammation was the same. Quinine was administered, and the patient began to improve immediately. February 5, the inflammation was gone, leaving a slight opacity of the cornea. One grain of quinine had been administered hourly.

*Case* II.—Æt. thirty-five, a conductor on a railroad. Patient had a severe keratitis of a week's standing when he consulted the doctor. He was directed to use atropine ointment and wear a bandage; but the inflammation increased during the following week. He was then administered quinine, and in six weeks he was well; the opacity had cleared away.

Inasmuch as these opacities are slow to clear, an early diagnosis becomes very important. He was persuaded that in malarial keratitis, the ulceration had a typical appearance, and he related other cases confirming his views.

Dr. W. T. Montgomery had also met a few characteristic cases of the disease under consideration, and besides employing ordinary local means, he had given four grains of quinine three times a day. The opacity had cleared.

Dr. E. L. Holmes had had the same class of patients, but he had been in the habit of treating all of them with quinine since his early days of practice. He also gave the same remedy in cases of chronic discharges from the ear, and in chronic affections generally. Of late, he had not given quinine quite so often, yet he prescribed it whenever local means failed, and in obstinate conjunctivitis. Some authors had discovered parasites in these diseases, but he had never observed them, and believed they might be secondary to the inflammation. The doctor reported a case of tetanus caused by a foreign body (a splinter of wood) in the orbit. October 12, 1881, a boy fifteen years old climbed up a scaffold six feet in height, and fell on a splinter of wood perpendicularly stuck in the ground. The splinter entered the left lower eyelid, and there was free bleeding from the nose. The doctor saw the patient a few days later, removed a piece of wood from the wound, and dressed it with a lotion of salicylic acid. The right eyelid began to inflame, and after a while was more involved than the left, which seemed partly restored, while the right eye was markedly exophthalmic. Patient had double vision. The wound discharged very little pus, but as much more flowed through the nostrils. After a week, the patient noticed that he had difficulty in opening his jaw, and the next day he had trismus, which increased in severity for a week longer, when Dr. Hotz saw the case. Deglutition was difficult, and fluid passed into the larynx. The conjunctiva was slightly œdematous, and the internal rectus muscle seemed affected. There was optic neuritis. By probing the wound, he felt a piece of wood extending to the right orbit, outward and backward, two inches deep; and a thin lamella of broken bone could also be felt in the wound. He succeeded in removing it, together with the piece of wood. Some improvement set in. The oxophthalmos decreased, but the improvement was only of a short duration. The next day the trismus was more pronounced, and the respiration became confined to one side. Patient died the next day in opisthotonos. The splinter had passed from one orbit into the other, through the left lachrymal bone, close to the cribriform plate. There was some emphysema of the orbits and of the face.

